# Antioxidant and Erythroprotective Effects of C-Phycocyanin from the Cyanobacterium *Spirulina* sp. in Attenuating Oxidative Stress Induced by Peroxyl Radicals

**DOI:** 10.3390/molecules31010169

**Published:** 2026-01-01

**Authors:** Cinthia Jael Gaxiola-Calvo, Diana Fimbres-Olivarría, Ricardo Iván González-Vega, Yaeel Isbeth Cornejo-Ramírez, Ariadna Thalía Bernal-Mercado, Saul Ruiz-Cruz, José de Jesús Ornelas-Paz, Miguel Ángel Robles-García, José Rogelio Ramos-Enríquez, Carmen Lizette Del-Toro-Sánchez

**Affiliations:** 1 Department of Research and Postgraduate in Food, University of Sonora, Blvd Luis Encinas y Rosales S/N, Col. Centro, Hermosillo 83000, Sonora, Mexico; a218213915@unison.mx (C.J.G.-C.); yaeel.cornejo@unison.mx (Y.I.C.-R.); thalia.bernal@unison.mx (A.T.B.-M.); saul.ruizcruz@unison.mx (S.R.-C.); 2 Department of Scientific and Technological Research, University of Sonora, Blvd Luis Encinas y Reforma S/N, Col. Centro, Hermosillo 83000, Sonora, Mexico; 3 Department of Health Sciences, University Center of the Valleys (CUVALLE), University of Guadalajara, Carr. a Guadalajara Km. 45.5, Ameca 46600, Jalisco, Mexico; ricardo.gonzalez@academicos.udg.mx; 4 Centro de Investigación en Alimentación y Desarrollo A.C.—Unidad Cuauhtémoc, Av. Río Conchos S/N, Parque Industrial, Ciudad Cuauhtémoc 31570, Chihuahua, Mexico; jornelas@ciad.mx; 5 Department of Medical and Life Sciences, Cienega University Center (CUCIÉNEGA), University of Guadalajara, Av. Universidad 1115, Lindavista, Ocotlán 47820, Jalisco, Mexico; miguel.robles@academicos.udg.mx; 6 Departamento de Ciencias Químico-Biológicas, Universidad de Sonora, Encinas y Rosales S/N Col. Centro, Hermosillo 83000, Sonora, Mexico; rogelio.ramos@unison.mx

**Keywords:** phycocyanin, *Spirulina* sp., hemolysis, antioxidant, erythroprotective, AAPH, peroxyl radical, in vitro digestion

## Abstract

Diseases caused by oxidative stress can present different susceptibilities depending on blood typing according to the ABO system and RhD factor, which turn out to be of great clinical importance. The use of antioxidants such as C-phycocyanin (a phycobiliprotein) could be an alternative to mitigate oxidative stress in the blood. Therefore, the objective of this study is to evaluate the antioxidant and erythroprotective activity of C-phycocyanin (C-PC) from *Spirulina* sp. against oxidative stress caused by peroxyl radicals, before and after in vitro digestion, comparing susceptibilities between blood groups. C-phycocyanin from *Spirulina* sp. was obtained commercially. The antioxidant capacity by ABTS+•, DPPH•, and FRAP assays of the bioaccessible fraction of C-PC increased compared to baseline in all assays. Samples appear to have high hydrogen atom transfer. C-PC is not cytotoxic in most blood groups. The AAPH hemolysis assays showed differences between blood groups, yielding results of 27.90, 22.60, 26.94, 27.66, 28.16, 28.34, and 24.91% hemolysis for O+, O−, A+, A−, B+, AB+, and AB−, respectively. Furthermore, in vitro digestion increased the erythroprotective effect in the bioavailable fraction in most blood groups, showing 37.12, 80.13, 5.48, 92.38, 67.93, 80.30, and 76.49% inhibition of hemolysis in O+, O−, A+, A−, B+, AB+, and AB−, respectively. These results demonstrate the biotechnological and biomedical potential of phycobiliproteins as safe candidates for the development of nutraceuticals and functional foods aimed at preventing oxidative damage.

## 1. Introduction

All aerobic organisms, including the human body, are harmed when exposed to higher than normal concentrations of oxygen. The peroxyl radical (ROO•) is a reactive oxygen species formed because of oxidative damage to lipids, DNA, and carbohydrates. Oxidative damage is continuous and, over time, may be linked to cellular disorders and the development of diseases [1]. Erythrocytes are particularly exposed to oxidative stress as they transport oxygen [2]. Therefore, antioxidants are required to prevent this oxidative stress, in this case could be C-phycocyanin (C-PC).

C-PC is a phycobiliprotein with a photosynthetic pigment function, present in various species of algae, cyanobacteria, and some cryptomonads. Based on their maximum absorbance, these light harvesting pigments are classified as allophycocyanin, phycocyanin, and phycoerythrin. This protein is a hexamer of monomers with two subunits containing chromophores: alpha (1 chromophore) and beta (2 chromophores), totaling 18 phycocyanobilin chromophores [3]. It is a natural blue colorant widely used in the food industry, which can also be incorporated as a functional ingredient due to its various health benefits. It has anti-inflammatory and anti-cancer properties, regulating intestinal flora, reducing intestinal permeability, increasing intestinal barrier function, as well as a remarkable antioxidant capacity, since it is able to neutralize free radicals, act directly on oxidative stress and inhibit lipid peroxidation [4,5,6]. Digestion releases biologically active chromopeptides from C-phycocyanin, whose activity is mainly related to the antioxidant potency provided by the chromophore [7].

Erythrocytes are anucleate cells that are essential components of the circulatory system due to their high capacity for oxygen transport, making them susceptible to oxidative damage. This type of damage can lead to lipid peroxidation of the membrane and cause hemolysis, resulting from the release of hemoglobin, which compromises their physiological function and the adequate supply of oxygen to the tissues [8,9]. Prolonged stress can induce morphological alterations in erythrocytes, affecting their flexibility and deformability, favoring the production of reactive oxygen species (ROS), and generating a state of oxidative stress. The increase in ROS can damage erythrocyte membranes through lipid peroxidation, compromising their structural and functional integrity and increasing their susceptibility to hemolysis [10,11]. Specifically, the peroxyl radical is generated during lipid peroxidation that occurs in cell membranes, initiating reactions with biological molecules such as carbohydrates, proteins, and nucleic acids. These interactions lead to the loss of membrane function and trigger significant biochemical and biophysical changes in cells and tissues. Consequently, this process is associated with various conditions related to inflammation, oxidative stress, lipid peroxidation, and multiple chronic degenerative diseases, including cardiovascular and neurological disorders, and the development of different types of cancer. Lipid peroxidation begins when a free radical removes a hydrogen atom from the CH_2_ group of a fatty acid, generating a lipid radical that subsequently reacts with molecular oxygen to form a peroxyl radical (ROO•) [12,13,14]. Damage to the erythrocyte membrane caused by the formation of peroxyl radicals can render the cell dysfunctional, leading to long-term disorders. For this reason, antioxidants such as C-PC are proposed to reduce or inhibit membrane damage caused by free radicals. The antioxidant capacity and antihemolytic compatibility of phycocyanin could vary according to blood group. The ABO blood group antigens play a relevant role in susceptibility to infections and the severity of various diseases, being related to a wide spectrum of pathologies, including metabolic diseases, autoimmune diseases, different types of cancer (such as ovarian, gastric and prostate cancer), as well as neuropsychiatric and rheumatological disorders, in addition to infectious conditions (*Plasmodium falciparum*, *Escherichia coli*, *Helicobacter pylori*, parvovirus B19, hepatitis B virus, chikungunya virus and others) [15,16,17,18,19].

Therefore, the objective of this research was to quantify the antioxidant activity of C-PC in erythrocytes of different blood groups and Rh (+/−) factors and in an in vitro digestion model. Currently, there are no studies comparing the erythroprotective capacity of C-PC from *Spirulina* sp. before and after in vitro digestion to observe its bioavailability.

## 2. Results

### 2.1. Quantification of the Antioxidant Capacity of Phycocyanin Before and After Digestion In Vitro

#### Antioxidant Capacity

The antioxidant activity provided by C-PC before and after in vitro digestion is summarized in Table 1. These different oxidation-reduction reactions allow for the quantification of the antioxidant capacity of samples, as is the case with the ABTS+•, DPPH•, and FRAP assays. The antioxidant capacity of the bioaccessible fraction increased compared to baseline in all assays. However, a slight decrease in DPPH and FRAP was observed in the bioavailable fraction, while ABTS increased. It is advisable to perform at least two antioxidant activity tests, as their physicochemical characteristics and the antioxidant mechanisms involved (hydrogen atom transfer HAT or single electron transfer SET) make it possible to identify variations that would otherwise go unnoticed [20,21,22,23]. In this case, FRAP only uses the SET mechanism, while DPPH and ABTS can use both depending on their reaction system. Based on the results obtained, the samples appear to have low electron transfer capacity, meaning they likely rely more on a HAT mechanism than a SET mechanism.

### 2.2. Cytotoxic and Erythroprotective Effects Before and After In Vitro Digestion

#### 2.2.1. Cytotoxicity of C-PC from the Cyanobacterium *Spirulina* sp. at Different Concentrations Before In Vitro Digestion

The cytotoxicity or blood biocompatibility test of C-PC of the cyanobacterium *Spirulina* sp. is applied to observe if there is toxicity from the protein when applied to erythrocytes, different concentrations were used for different blood groups (Table 2). According to the results, C-PC is not cytotoxic in most blood groups. However, no cytotoxicity was observed in blood groups O− and B+ at the maximum concentration (300 µg/mL). In the remaining blood groups, a slight increase was observed starting at 125 µg/mL, with AB+, AB−, A+, and O+ being the most susceptible. Generally, Rh-negative blood groups are less susceptible to cytotoxicity. However, compared with AAPH, that was used as positive control, the cytotoxicity of P-CP was almost nil.

#### 2.2.2. Erythroprotection of C-Phycocyanin from the Cyanobacterium *Spirulina* sp. at Different Concentrations After an In Vitro Digestion

**Figure 1:**
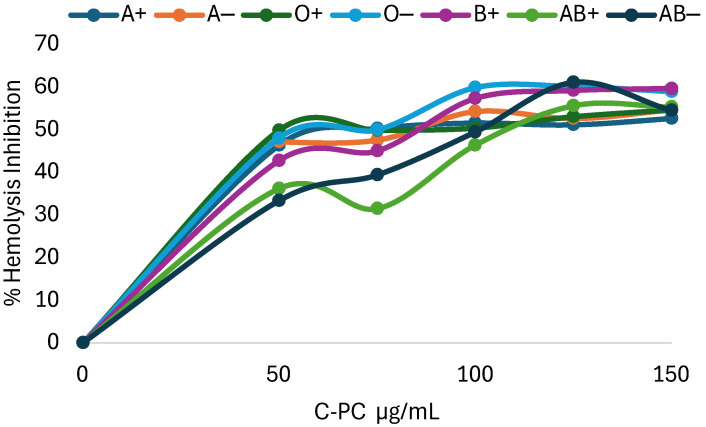
Percentage of hemolysis inhibition using C-phycocyanin at different concentrations on erythrocytes supplemented with AAPH.

Inhibition of hemolysis caused by peroxyl radicals induced by the azocompound AAPH at different concentrations of C-PC. Different responses were obtained in different blood groups and Rh factors. C-PC can inhibit the formation of peroxyl radicals in a differentiated manner in blood groups. Blood type O− requires a lower concentration than the other groups for radical inhibition. In contrast, type AB− requires a higher concentration of C-PC to achieve the same result, as can be seen in Figure 1.

Since C-PC acts differently in each blood group and Rh factor, the inhibitory concentration 50 (IC50) was estimated using equations obtained from the graphs, yielding the results in Table 3, where it was found that O− requires the lowest concentration of C-PC to inhibit 50% of hemolysis against the azo compound AAPH, followed by type AB+, which has a different relationship with the azo compound compared to AB−, which requires the highest concentration of C-PC to achieve the same result.

#### 2.2.3. Cytotoxic Effect of IC50 of C-PC After In Vitro Digestion

**Figure 2:**
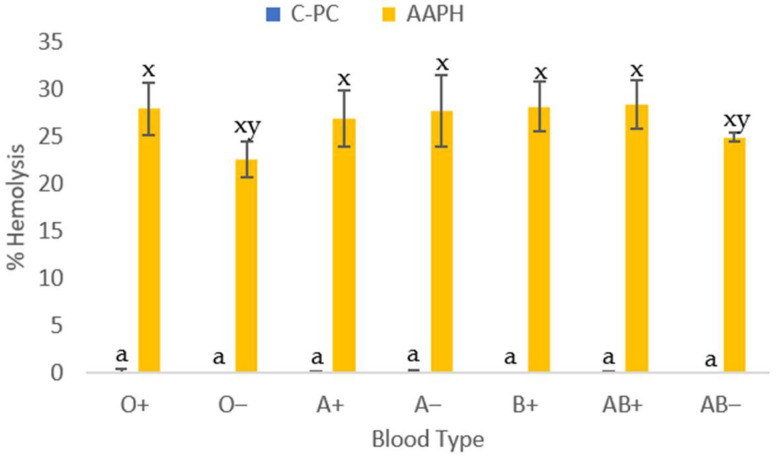
Blood cytotoxicity assay on human erythrocytes of different blood types (ABO and RhD factor), applying the inhibitory concentration 50 (IC50) of C-phycocyanin obtained from the cyanobacterium *Spirulina* sp. Data are presented as the mean ± SD (standard deviation) of at least three replicates (*n* ≥ 3). Distinct lowercase letters (x, y) represent significant differences in the effect of AAPH (40 mM) between blood groups (*p* < 0.001). Same lowercase letter (a) represent no significant differences in the IC50 of C-PC between blood groups (*p* > 0.001).

The analysis provides information on the biocompatibility of C-phycocyanin from the cyanobacterium *Spirulina* sp. solubilized in physiological solution and applied to erythrocytes of different blood groups and Rh factors (Figure 2). This study was conducted to verify that C-PC at specific IC50 levels did not cause damage to erythrocytes. The results showed that it did not cause damage at these concentrations, compared to AAPH administered at the same IC50 levels.

#### 2.2.4. Cytotoxic Effect of C-PC Before In Vitro Digestion

**Figure 3:**
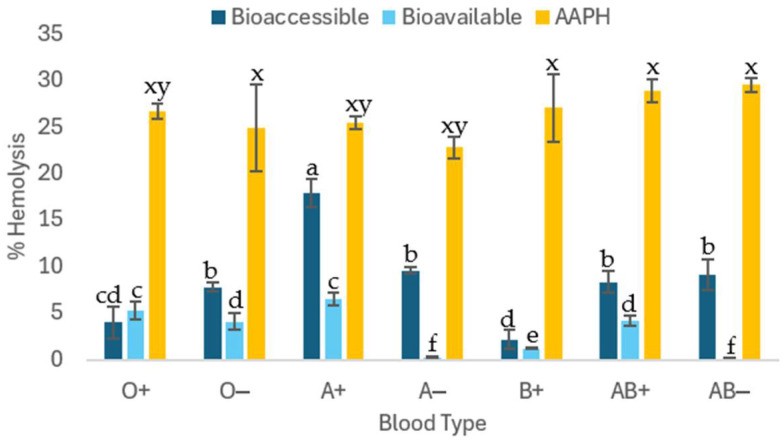
Blood cytotoxicity assay in human erythrocytes of different ABO blood groups, using 1 mg/mL of C-phycocyanin obtained from the cyanobacterium *Spirulina* sp. after in vitro digestion. Bioaccessible: sample inside the cellulose membrane and Bioavailable: sample outside the cellulose membrane. Different lowercase letters (x, y) represent significant differences in the AAPH (40 mM) positive control between blood groups, and lowercase letters (a, b, c, d, e, f) indicate significant differences between in vitro digestion fractions and blood groups (*p* < 0.005).

The blood biocompatibility test shows cytotoxicity results of the compound on human erythrocytes. In this case, an in vitro digestion of 1 mg/mL of C-PC was performed on the different ABO blood groups (Figure 3). Group O− showed more hemolysis in the bioaccessible fraction, followed by AB+ and AB−. Approximately 30% of C-PC is bioavailable and, instead of causing toxicity, it will help protect erythrocytes from damage, principally A− and AB− groups.

### 2.3. Erythroprotection Assays Before and After In Vitro Digestion

#### 2.3.1. Erythroprotection of C-PC Using Its IC50 Before In Vitro Digestion

Table 4 shows the determination of the median inhibitory concentration (IC50) of C-PC in erythrocytes of different blood groups, which is the concentration required to inhibit 50% of damage or hemolysis. Blood groups A− and O+ require the least amount of C-PC to inhibit 50% of hemolysis, while groups AB+ and AB− require larger quantities.

#### 2.3.2. Erythroprotective Effect of C-Phycocyanin After In Vitro Digestion

**Figure 4:**
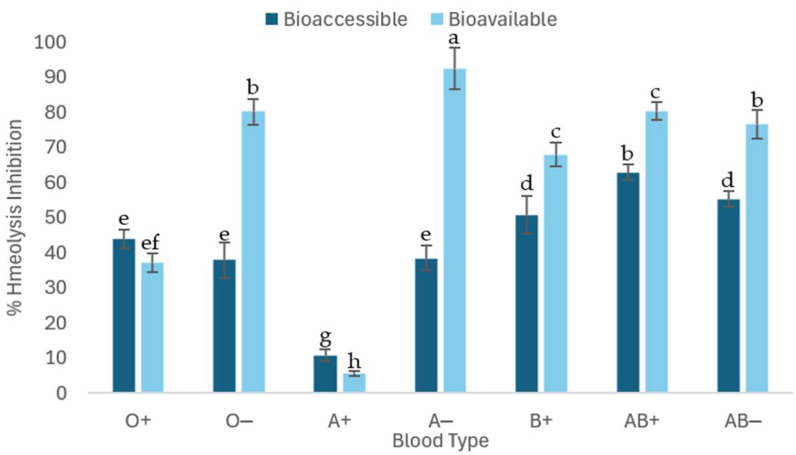
Erythroprotection assay against hemolysis induced by the azo compound AAPH (40 mM) inhibited by 1 mg/mL of C-PC after in vitro digestion in erythrocytes of different ABO blood groups. Bioaccessible + AAPH: sample inside the cellulose membrane against peroxyl radicals. Bioavailable + AAPH: sample outside the cellulose membrane against peroxyl radicals. Different lowercase letters indicate significant differences between in vitro digestion fractions and blood groups (*p* < 0.005).

The erythroprotective assay, evaluated after in vitro digestion of C-PC at a concentration of 1 mg/mL, showed that it was able to differentially inhibit peroxyl radicals generated by the azo compound AAPH (40 mM) among the different ABO blood groups and RhD factors. As shown in Figure 4, significant differences were observed between the bioavailable and bioaccessible fractions in all blood groups. In Rh positive groups, the differences in the percentage of hemolysis inhibition between the two fractions were smaller compared to Rh negative groups. In general, the bioavailable fraction showed a higher percentage of hemolysis inhibition in most blood groups, except for types O and A+, in which the bioaccessible fraction showed greater inhibitory capacity. The values obtained were variable, with inhibition percentages ranging from 5% to 90% depending on the blood group and the type of fraction. These variations could be explained by the differential affinity of the compounds towards the components of the erythrocyte membrane, particularly by the variability in the terminal sugars characteristic of each blood type.

### 2.4. Damage to Erythrocytes Observed Using Immersion Optical Microscopy

Images were taken using an optical microscope of erythrocytes added with samples of Triton × 1%, AAPH, and C-PC before and after in vitro digestion to observe the morphological damage caused to the erythrocytes.

#### 2.4.1. Microscope Images of Blood Type O+

**Figure 5:**
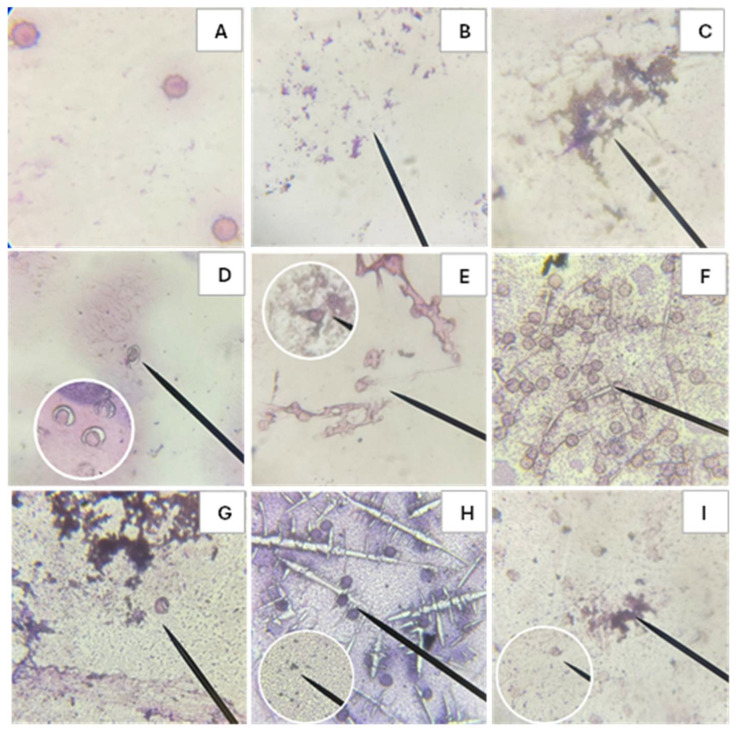
Smears from biocompatibility and hemolysis inhibition tests before and after in vitro digestion of C-phycocyanin (C-PC) from the cyanobacterium *Spirulina* sp. in blood group O+. Where (**A**) Negative control, (**B**) Positive control Triton 1%, (**C**) Positive control AAPH, (**D**) C-PC, (**E**) C-PC + AAPH, (**F**) Fraction inside the cellulose membrane after in vitro digestion of the C-PC sample, (**G**) Fraction within the cellulose membrane after in vitro digestion of the C-PC + AAPH sample, (**H**) Fraction outside the cellulose membrane after in vitro digestion of the C-PC sample, (**I**) Fraction outside the cellulose membrane after in vitro digestion of the C-PC + AAPH sample.

Microscopic observation provides visual evidence of the morphological changes occurring in erythrocytes of blood type O+ after exposure to the different treatments shown in Figure 5. Figure 5A depicts healthy erythrocytes with intact cell membranes, whereas Figure 5B illustrates complete hemolysis produced by Triton, used as the positive control of hemolysis. AAPH induces both hemolysis and oxidative damage to red blood cells, as observed in Figure 5C. In contrast, C-PC does not exhibit cytotoxic effects (Figure 5D). A comparison between Figure 5C,E shows that C-PC confers protective activity against AAPH-induced damage, as indicated by the presence of erythrocytes retaining their normal morphology. Furthermore, the in vitro digestion products demonstrate that both the bioavailable and bioaccessible fractions do not induce morphological alterations in erythrocytes (Figure 5F,H) and can mitigate AAPH-induced damage, as evidenced by the presence of healthy cells (Figure 5G,I).

#### 2.4.2. Microscope Images of Blood Type O−

**Figure 6:**
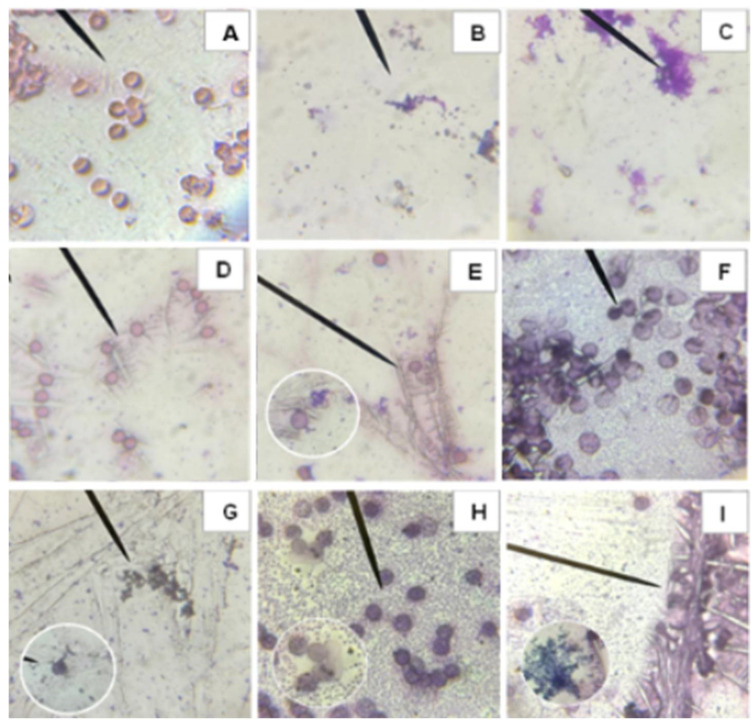
Smears from biocompatibility and hemolysis inhibition tests before and after in vitro digestion of C-phycocyanin (C-PC) from the cyanobacterium *Spirulina* sp. in blood group O−. Where (**A**) Negative control, (**B**) Positive control Triton 1%, (**C**) Positive control AAPH, (**D**) C-PC, (**E**) C-PC + AAPH, (**F**) Fraction inside the cellulose membrane after in vitro digestion of the C-PC sample, (**G**) Fraction within the cellulose membrane after in vitro digestion of the C-PC + AAPH sample, (**H**) Fraction outside the cellulose membrane after in vitro digestion of the C-PC sample, (**I**) Fraction outside the cellulose membrane after in vitro digestion of the C-PC + AAPH sample.

Similarly to Figure 5, microscopic evaluation of O− was performed, allowing visualization of the morphological alterations in erythrocytes after the application of the different samples shown in Figure 6. Figure 6A shows intact erythrocytes with undamaged membranes, while Figure 6B shows completely hemolyzed cells due to the Triton treatment, used as a positive control. AAPH causes hemolysis and oxidative stress in red blood cells, as seen in Figure 6C. In contrast, C-PC does not show cytotoxic effects (Figure 6D). Comparing Figure 6C,E, it can be observed that C-PC exerts a protective effect against AAPH-induced damage, since erythrocytes with healthy morphology can still be distinguished. Furthermore, in vitro digestions indicate that both the bioavailable and bioaccessible fractions do not generate morphological alterations in erythrocytes (Figure 6F,H) and contribute to reducing the damage caused by AAPH, with healthy cells evident in Figure 6G,I.

## 3. Discussion

### 3.1. Quantifying the Antioxidant Capacity of C-Phycocyanin Before and After Digestion In Vitro Digestion

#### Antioxidant Capacity

**Figure 7:**
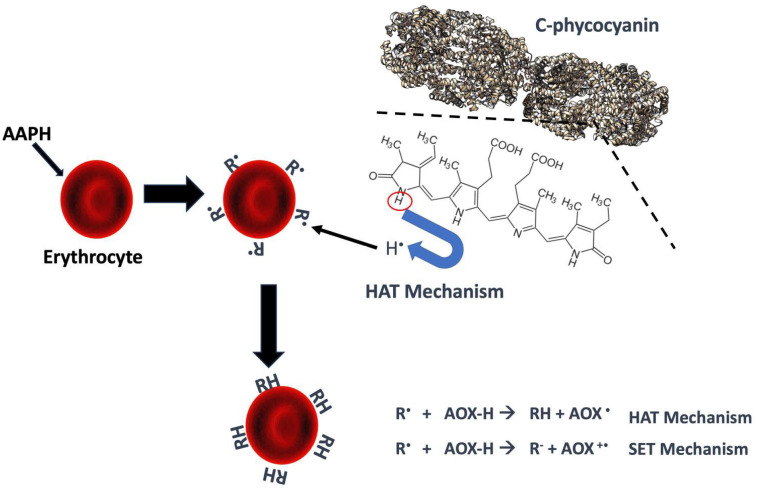
Proposed antioxidant and erythroprotective mechanisms for c-phycocyanin. R^•^: Radical, RH: Radical reduced (neutralized), AOX: Antioxidant. HAT: Hydrogen atom transfer, SET: Single Electron Transfer. The structure of C-phycocyanin was obtained from CHimeraX (PDB: 1GH0).

The results in Table 1 indicate that C-PC exhibits antioxidant activity in the ABTS+• assay. The variations observed in its antioxidant capacity depending on the method used can be attributed to the different SET (Single Electron Transfer) and HAT (Hydrogen atom transfer) mechanisms that characterize each of the tests applied. Compared to DPPH•, the ABTS radical is more sensitive to hydrophilic compounds, such as soluble polyphenols and some water-soluble pigments (phycobiliproteins, oxygenated carotenoids). In the ABTS+• assay, the bioavailable fractions of C-PC showed slightly higher activity than the bioaccessible fractions in two of the three digestions. On the other side, in the DPPH• assay, the opposite behavior was observed, with the bioavailable fractions showing lower antioxidant activity than the bioaccessible fractions. This is probably because not all antioxidant compounds released during digestion are able to cross the simulated intestinal membrane (mainly larger compounds or those associated with complex matrices). The FRAP assay showed the same trend as DPPH•, with bioavailable fractions exhibiting lower antioxidant capacity than bioaccessible fractions. The fact that both DPPH• and FRAP (assays mainly associated with the SET mechanism), show similar results could indicate a low contribution of this mechanism in bioavailable fractions, hence, the HAT mechanism being the most predominant (Figure 7). This suggests that compounds capable of transferring electrons (such as certa mecin phenolic acids and flavonoids) may not be absorbed efficiently or may be degraded during gastrointestinal transit.

### 3.2. Cytotoxic and Erythroprotective Effects Before and After In Vitro Digestion

#### 3.2.1. Cytotoxic Effect of IC50 of C-Phycocyanin

When using the IC50 concentrations obtained from the equations corresponding to each blood group, cytotoxicity below 5% hemolysis was observed in all blood groups studied. On the other hand, the azo compound AAPH presented an average cytotoxicity of 27.48 ± 3.8% (Table 2). Evaluating the cytotoxicity results, it was found that C-phycocyanin extract is harmless in the seven blood groups studied when applying the IC50, since the critical hemolysis limit for good blood biocompatibility must be less than 5% according to ISO 10993-4 [24]. In the case of AB+, the hemolytic damage presented is considered minimal.

#### 3.2.2. Cytotoxic Effect of C-Phycocyanin After In Vitro Digestion

In the case of the bioaccessible fraction, cytotoxic results exceeding the permitted 5% hemolysis were obtained, with the exception of groups O and B+, which were within the biocompatibility values. In the case of the bioavailable fraction, C-PC is only cytotoxic in blood group A+, with a value above that permitted by ISO 10993-4. As a result, the C-PC compound is harmless after in vitro digestion, except for blood type A+, inferring that digestion helps the absorbed compound to be biocompatible with red blood cells and the unabsorbed fraction is cytotoxic. The azo compound AAPH showed cytotoxicity with significant differences between blood groups, with an average hemolysis of 27.18 ± 2.9% (Figure 3). Control of hemolysis caused by peroxyl radicals produced by the azo compound AAPH showed cytotoxicity with an average hemolysis of 27.18 ± 2.9%.

#### 3.2.3. Erythroprotection of C-PC Using Its IC50 Prior to In Vitro Digestion

The IC50 of C-PC obtained from the equations was confirmed, as it inhibits half of the damage caused by the azo compound AAPH. Concentrations ranging from 82.66 to 104.37 µg/mL of C-PC were applied. This variation could be caused by the different conformation of the blood group membrane, which contain different terminal sugars or antigens that can interfere with the action of compounds in terms of both antioxidant capacity and sensitivity to hemolysis, in this case of C-PC or AAPH, respectively (Table 4). C-PC stood out in the erythroprotective effect determinations to obtain IC50 because values close to this were reached with relatively low concentrations. C-PC has a potent antioxidant effect thanks to its phycocyanobilin chromophores. The main mechanism of cell damage associated with AAPH is the generation of free radicals. Phycocyanin is highly efficient at inhibiting radicals through tetrapyrrole chromophore groups (phycocyanobilin) that react quickly with ROO• radicals, neutralizing them before they initiate chain reactions and prevent lipoperoxidation. Although the potential of phycocyanin is evident, there is some variation in the concentrations required to reach the IC50, due to the different interactions between C-PC and the different compositions of erythrocyte membranes and blood types [25,26,27].

#### 3.2.4. Erythroprotective Effect of C-Phycocyanin After In Vitro Digestion

According to Figure 4, the bioaccessible fraction shows inhibitions of up to 62.87% with significant differences depending on blood type. In contrast, the bioavailable fraction shows hemolysis inhibition of up to 92.38% with significant differences depending on blood type, with both fractions inhibiting AAPH-induced radicals. The bioaccessible fraction has a significantly lower hemolysis inhibitory capacity than the bioavailable fraction. The erythroprotective assay of C-PC against AAPH-induced hemolysis after in vitro digestion, specifically the bioaccessible fraction samples, showed that there are no significant differences in each blood group except for O-, where hemolysis inhibition does not differ between samples. However, there are significant differences between the bioavailable fraction and its % hemolysis inhibition between blood groups when applying the same fraction. The erythroprotective assay against hemolysis caused by AAPH of C-PC after in vitro digestion, specifically of the bioavailable fraction, shows that the cytotoxicity of AAPH resulted in an average of 27.18 ± 2.9% hemolysis. The bioavailable fractions of C-PC inhibiting between 40 and 90% of hemolysis depending on the blood group.

The ABO and Rh blood groups are differentiated by the presence of specific antigens on the surface of red blood cells. As demonstrated in the results, C-phycocyanin interacts differently with these antigens, affecting its erythroprotective efficacy. It is hypothesized that C-phycocyanin may have specific affinities for blood groups due to its structural properties and electrostatic charge. This explains why the effectiveness of C-phycocyanin varies with different blood groups (A, B, AB, and O), with a more pronounced effect observed in one group compared to another. In the case of Rh groups, the difference between positive and negative lies in the presence of the D antigen on the surface of red blood cells. C-phycocyanin interacts differently with Rh positive erythrocytes due to the presence of this antigen, which modulates its antioxidant and protective effect. However, further experimental studies directly evaluating these interactions and including diverse population samples are essential to confirm these findings.

## 4. Materials and Methods

### 4.1. Reagents

ABTS [2,2-azino-bis-(3-ethylbenzothiazoline-6-sulfonic acid] powder, DPPH [2,2-diphenyl-1-picrylhydrazyl] powder, Trolox [6-hydroxy-2,5,7,8-tetramethylchroman-2-carboxylic acid] powder, TPTZ [2,4,6-tripridyl-s-triazine] powder, Tris-HCl biological buffer, AAPH [2,2-azobis(2-methylpropionamidine) dihydrochloride] powder or granules and C-phycocyanin from *Spirulina* sp. (lyophilized powder were purchased from Sigma-Aldrich (St. Louis, MO, USA). All other chemicals and solvents were of the highest commercial quality.

### 4.2. Quantification of the Antioxidant Capacity of C-Phycocyanin Before and After In Vitro Digestion

The antioxidant activity of C-PC obtained from the cyanobacterium *Spirulina* sp. was evaluated pre and post digestion in vitro, analyzing both its antiradical capacity (ABTS+• and DPPH•) and its reduced power (FRAP). To quantify the results, a Trolox standard curve was prepared, and the values of the different methods are expressed as µmol ET (Trolox equivalents)/g DW. Trolox is a synthetic hydrophilic antioxidant standard analogous to vitamin E. The Trolox equivalent unit implies the antiradical and reduced capacity of the Trolox antioxidant. In addition, the ability to inhibit peroxyl radicals generated by AAPH on human erythrocytes was analyzed [28].

#### 4.2.1. Assay to Evaluate the Free Radical Inhibition Capacity of 2,2-Azinobis-(3-ethylbenzothiazolin)-6-sulfonic Acid (ABTS+•)

To quantify the antiradical activity against the ABTS+• free radical, the previously described technique was used, with some modifications [29]. To generate the radical, 19.3 mg of ABTS were dissolved in 5 mL of distilled water, and then 88 μL of a potassium persulfate solution (K_2_S_2_O_8_, 0.0378 g/L) was added. The mixture was allowed to stand in the dark for 16 h to allow the formation of ABTS+•. This solution was then diluted with 99% ethanol until the absorbance was adjusted to 0.7 ± 0.05 at 734 nm. After the allotted time, the following treatments were placed in triplicate in a 96-well microplate: negative control (20 µL of ethanol + 270 µL of ABTS+•), standard control (20 µL of the standard antioxidant + 270 µL of ABTS+•), and the samples (20 µL of the extract + 270 µL of ABTS+•). The samples were left to stand for 30 min in the dark at room temperature, after which readings were taken at 734 nm in a microplate spectrophotometer (Multiskan Go, Thermo Scientific, Waltham, MA, USA). The results were reported as µmol ET (Trolox equivalents)/g DW.

#### 4.2.2. Assay to Evaluate the Free Radical Inhibition Capacity of 1,1-Diphenyl-2-picrylhydrazyl (DPPH•)

The ability to inhibit the DPPH• free radical was assessed following the previously described protocol [30]. A methanolic solution of DPPH• (6 × 10^−5^ mol·L^−1^) was prepared, and the absorbance was subsequently adjusted to 0.7 ± 0.05 at 515 nm by adding 99% methanol. The following treatments were performed in triplicate on a 96-well microplate: negative control (20 μL of methanol + 200 μL of DPPH•), standard control (20 μL of the standard antioxidant + 200 μL of DPPH•), and the samples (20 μL of the extract + 200 μL of DPPH•). The samples were left to stand for 30 min at room temperature in the dark. The absorbances were read at 515 nm in a spectrophotometer (Multiskan Go, Thermo Scientific, Waltham, MA, USA). The results were reported as µmol ET (Trolox equivalents)/g DW.

#### 4.2.3. Evaluation of the Reducing Capacity of Ferric Ions (Fe^3+^) Using the FRAP Test (Ferric Ion Reducing Antioxidant Power)

The reducing power was evaluated following the previously described methodology, with some modifications [31]. Three stock solutions were prepared: a sodium acetate buffer (300 mM, pH 3.6), a ferric chloride solution (FeCl_3_, 20 mM), and a TPTZ (2,4,6-triphenyl-s-triazine, 10 mM) solution in HCl (40 mM). The working mixture for the FRAP assay was prepared by combining these solutions in a 10:1:1 ratio (buffer:FeCl_3_:TPTZ). In a 96 well microplate, the following treatments were added in triplicate: negative control (20 µL of methanol + 280 µL of FRAP reagent), standard control (20 µL of standard antioxidant + 280 µL of FRAP) and the samples (20 µL of the extract + 280 µL of FRAP). The samples were left to stand for 30 min in the dark at room temperature. The optical densities at 638 nm were obtained using a spectrophotometer (Multiskan Go, Thermo Scientific, Waltham, MA, USA). The results were reported as µmol ET (Trolox equivalents)/g DW.

### 4.3. In Vitro Digestion

This was carried out using an established protocol, with modifications [32]. Human amylase (saliva) was added to the sample and left to stand for 2 min. After this time, physiological solution was added, followed by homogenization. It was then acidified to pH 2 with 6 M HCl. Once adjusted, pepsin at 315 U/mL was added, and it was placed in a water bath for 2 h at 37 °C and 80 rpm. After this time, the pH was neutralized to 7.4 with 1.25 M NaHCO3, 4 mg/mL pancreatin was added, homogenized, and previously hydrated cellulose membranes were filled with physiological solution for 1 day, placed in a water bath for 4 h at 37 °C and 80 rpm. After the incubation period, the antioxidant capacity was quantified (Table 5).

### 4.4. Cytotoxic and Erythroprotective Effect

#### 4.4.1. Erythrocyte Cytotoxicity Assay

The assays performed using human erythrocytes (RBC) were conducted in accordance with Mexican regulations (NOM-253-SSA1-2012) and international standards (FDA: CFR Code of Federal Regulations Title 21, Part 640, Additional Standards for Human Blood and Blood Products, Support. B Red Blood Cells, Sec. 640.14, Blood Tests (21CFR640.14)). The protocol was approved by the ethics committee of the General Hospital of the State of Sonora (CI 2023-47). Informed consent was obtained from all donors.

Red blood cell (RBC) samples were collected by venipuncture into EDTA tubes using the Vacutainer system. Blood was obtained from all possible groups (ABO with Rh+ and −) from apparently healthy individuals over the age of 18 with their respective informed consent. Washing was performed with physiological solution (PS) by adding 3000 µL to 1000 µL of blood, which was then centrifuged at 1500 rpm for 10 min. After this time, the supernatant was removed, and the process was repeated three times to remove all plasma. Subsequently, a 2% erythrocyte suspension was made from all blood groups obtained (using 0.9% physiological solution). Positive control (100 µL erythrocyte suspension + 100 µL physiological solution + 100 µL Triton 1%), negative control (100 µL erythrocyte suspension + 200 µL physiological solution), sample (100 µL erythrocyte suspension + 100 µL physiological solution + 100 µL sample). Subsequently, the controls and tubes with the samples were incubated for 3 h at 37 °C, the reaction sample was diluted with 1 mL of physiological solution and centrifuged at 7000 rpm for 2 min. Three hundred µL of the supernatant was taken and read in triplicate in a spectrophotometer with a microplate reader at 540 nm. The cytotoxic effect was measured by quantifying the percentage of hemolysis according to Equation (1):

(1) $$\%\;of\;hemolysis=\frac{A_{sample}-A_{PBS}}{A_{Triton}-A_{PBS}}\times 100$$

#### 4.4.2. Erythroprotection Assay, Quantifying Antihemolytic Activity in Erythrocytes by 2,2′-Azobis-(2-methylpropionic acid-(2-methylpropionamidine) (AAPH)

Oxidative hemolysis was induced using AAPH, a compound recognized as a free radical initiator, following previously described methods [33,34]. The following treatments were prepared: negative control (100 μL of 2% erythrocyte suspension + 200 μL of physiological saline), positive control (C+) (100 μL of erythrocyte suspension + 100 μL of physiological saline + 100 μL of AAPH), and the sample treatment (100 μL of erythrocyte suspension + 100 μL of AAPH + 100 μL of sample). The tubes were incubated at 37 °C with agitation at 40 rpm in the dark for 3 h. After incubation, the reaction sample was diluted with 1 mL of physiological solution and centrifuged at 7000 rpm for 2 min. The absorbance of the supernatant was measured at 540 nm in a spectrophotometer using 96-well microplates. All measurements were performed in triplicate. The percentage of hemolysis inhibition was calculated using Equation (2):

(2) $$\%\;Inhibition\;of\;hemolysis=\frac{\left(A_{C+})-(Asample-A_{PBS}\right)}{A_{C+}}\times 100$$

### 4.5. Erythrocyte Cell Membrane

To examine alterations in the erythrocyte cell membrane, an optical microscope (Eclipse FN1 model with 100x magnification) was used. In this analysis, the supernatants were evaluated immediately after reading. Approximately 50 µL of fresh plasma was added to the compacted erythrocytes obtained in the hemolysis inhibition assay, mixing carefully to avoid mechanical damage to the membranes. A drop of blood suspension was added to a slide to form a thin layer of erythrocytes. Wright’s stain was used to examine the integrity of their membranes. Micrographs were analyzed at 100x magnification and are presented with a 5 µm scale bar to facilitate cell size comparison. Images were acquired using NIS-Elements F v4.11.0 software (Nikon Instruments Inc., Americas, New York, NY, USA; https://www.microscope.healthcare.nikon.com/es_AMS/products/software/nis-elements/viewer, accessed on 13 May 2025).

## 5. Statistical Analysis

Experimental results were evaluated using inferential statistics with a 95% confidence level. All trials were performed with at least three independent replicates (*n* ≥ 3), and data were reported as mean ± standard deviation (SD). To determine significant differences among treatments, blood groups, and fractions (bioaccessible/bioavailable), a one-way analysis of variance (one-way ANOVA) was applied. Data processing and statistical analysis were conducted using specialized software, and the interpretation was based on comparisons among ABO/Rh blood groups, fractions before and after in vitro digestion, as well as on the interaction between C-phycocyanin, the peroxyl radical generated by AAPH, and the erythrocyte membrane.

## 6. Conclusions

Analysis of the erythroprotective effect of C-phycocyanin from the cyanobacterium *Spirulina* sp. suggests a possible relationship between different blood antigens (ABO and RhD) and their antihemolytic action. After in vitro digestion, the results indicate that the bioaccessible fractions exhibit a higher percentage of inhibition of peroxyl radical-induced hemolysis. In particular, the digested fraction of C-phycocyanin shows significantly greater inhibition compared to the undigested samples. These findings suggest that oral administration of C-phycocyanin is an effective route to exert beneficial health effects and inhibit the formation of peroxyl radicals, thereby protecting the erythrocyte membrane. It is most beneficial for blood groups O−, A−, B+, AB+, and AB−, exceeding 70% inhibition of hemolysis. The intake of C-phycocyanin could be incorporated into a food matrix to increase its nutritional value. The optical differences between treatments are noticeable under the microscope, where the cytotoxic activity of AAPH and erythroprotection of the samples can be observed. These findings highlight potential biotechnological and biomedical applications of the extract as a safe ingredient in the development of nutraceuticals and functional foods, as well as in formulations with preventive potential against oxidative stress.

## Figures and Tables

**Table 1:** Determination of antioxidant activity of C-PC before and after in vitro digestion (µmol ET/g DW).

Sample	ABTS+•	DPPH•	FRAP
C-PC before digestion in vitro	1.58 Ba ± 0.42	1.32 ABa ± 0.08	0.03 Cb ± 0.02
Bioaccessible Fraction	6.97 Aa ± 0.42	1.21 bA ± 0.48	0.13 Ac ± 0.01
Bioavailable Fraction	7.34 Aa ± 0.08	0.89 Ab ± 0.42	0.07 Bc ± 0.01

Data are presented as mean ± SD (standard deviation) from at least three replicates (*n* ≥ 3). A one-way analysis of variance (ANOVA) was performed. Different uppercase letters represent significant sample differences for each sample or fraction between rows (*p* < 0.001). Different lowercase letters represent significant bioassay differences between columns (*p* < 0.001).

**Table 2:** Percentage of hemolysis caused by C-phycocyanin from the cyanobacterium *Spirulina* sp. at different concentrations on human erythrocytes in different blood types and Rh factors.

	µg/mL						
ABO Rh +/− *	50	75	100	125	150	300	AAPH
A+	0 A ± 1.35	0 A ± 1.10	0.58 A ± 0.59	0.29 A ± 0.54	2.26 A ± 1.58	1.47 B ± 0.47	25.13 ABC ± 2.53
A−	0 B ± 0.21	0.12 A ± 0.77	0 A ± 0.78	0 A ± 1.68	0 B ± 1.66	0.65 B ± 1.44	20.95 E ± 0.96
O+	0 A ± 0.97	0.03 A ± 1.26	0 A ± 0.83	0.34 A ± 0.85	0.04 B ± 0.81	1.13 A ± 0.94	27.01 AB ± 0.49
O−	0 A ± 0.89	0 A ± 0.39	0 A ± 0.37	0 A ± 0.93	0 B ± 0.91	0 B ± 1.31	26.23 BC ± 0.97
B+	0.97 A ± 0.75	0.43 A ± 0.95	0 A ± 0.44	0 A ± 0.35	0 B ± 1.41	0.98 A ± 2.03	25.29 C ± 0.55
AB+	0 A ± 0.73	0.25 A ± 0.90	0 A ± 0.51	0 A ± 2	1.01 B ± 0.61	2 A ± 0.5	23.35 D ± 1.24
AB−	0 A ± 0.77	0 A ± 0.50	0 A ± 0.91	0.70 A ± 0.39	0.28 B ± 0.95	1.75 AB ± 0.30	28.10 A ± 1.00

* ABO Rh +/−: Refers to the blood type and its Rh factor. AAPH: 40 mM. A one-way analysis of variance (ANOVA) was performed. Different uppercase letters represent significant sample differences for each sample or fraction (*p* < 0.001).

**Table 3:** Inhibitory concentration 50 in µg/mL of C-phycocyanin against hemolysis caused by peroxyl radicals obtained by equations.

ABO Rh +/− *	C-PC µg/mL	Equation	R^2^	Model
A+	82.66	y = 0.5216x + 6.8853	0.9593	Linear
A−	95.25	y = −0.0055x^2^ + 1.0478x + 1.0108	0.9352	Polynomial
O+	92.53	y = 0.5512x − 1.0054	0.9896	Linear
O−	82.05	y = −0.0072x^2^ + 1.1813x + 1.5912	0.9316	Polynomial
B+	88.26	y = 0.4973x + 6.1085	0.9677	Linear
AB+	104.37	y = −0.0057x^2^ + 1.1897x + 1.3272	0.9704	Polynomial
AB−	101.94	y = −0.0051x^2^ + 1.0397x + 0.0859	0.9986	Polynomial

* ABO Rh +/−: Refers to the blood type and its Rh factor.

**Table 4:** Percentage inhibitor of AAPH 40 Mm induced hemolysis using the IC50 of C-PC.

Bood Type	µg/mL	% Hemolysis Inhibition
A+	82.66	50.08 ± 1.7
A−	95.25	48.58 ± 1.9
O+	92.53	50.07 ± 1.3
O−	82.05	50.70 ± 0.7
B+	88.26	50.96 ± 1.1
AB+	104.37	50.27 ± 1.9
AB−	101.94	49.95 ± 1.7

**Table 5:** Quantities of samples or reagents to be added to the in vitro digestion.

In Vitro Digestion	Sample or Reagent	Volume	Concentration
C-phycocyanin	C-PC	0.5 mL	1 mg/mL
	α-amylase	1 mL	Human origin
	Physiological solution	1 mL	0.9%
	Pepsin	1 mL	315 U/mL
	Pancreatin	700 µL	4 mg/mL

## Data Availability

The original contributions of data presented in this research are included in the article; further inquiries can be directed to the corresponding authors.

## Abbreviations

The following abbreviations are used in this manuscript:

AAPH	2,2-Azobis(2-methylpropionamidine) dihydrochloride
ABTS	2,2-azino-bis-(3-ethylbenzothiazoline-6-sulfonic acid
APC	Allophycocyanin
B-PE	B-Phycoerythrin
C-PC	C-Phycocyanin
DPPH	2,2-diphenyl-1-picrylhydrazyl
DW	Dry Weight
FRAP	Ferric Reducing Antioxidant Power
PBP	Phycobiliprotein
PSU	Practical Salinity Units
PS	Physiological Solution
R-PC	R-Phycocyanin
TPTZ	2,4,6-Tripridil-s-triazine
Trolox	6-hydroxy -2,5,7,8-tetramethylchroman-2-carboxylic acid
